# Supplementary figures and images for: Plasma Metabonomics in Insulin-Resistant Hypogonadic Patients Induced by Testosterone Treatment
Source: Int J Mol Sci. 2022 Jul 14;23(14):7754. doi: 10.3390/ijms23147754 (PMC9324383; doi:10.3390/ijms23147754)

## Slide 1
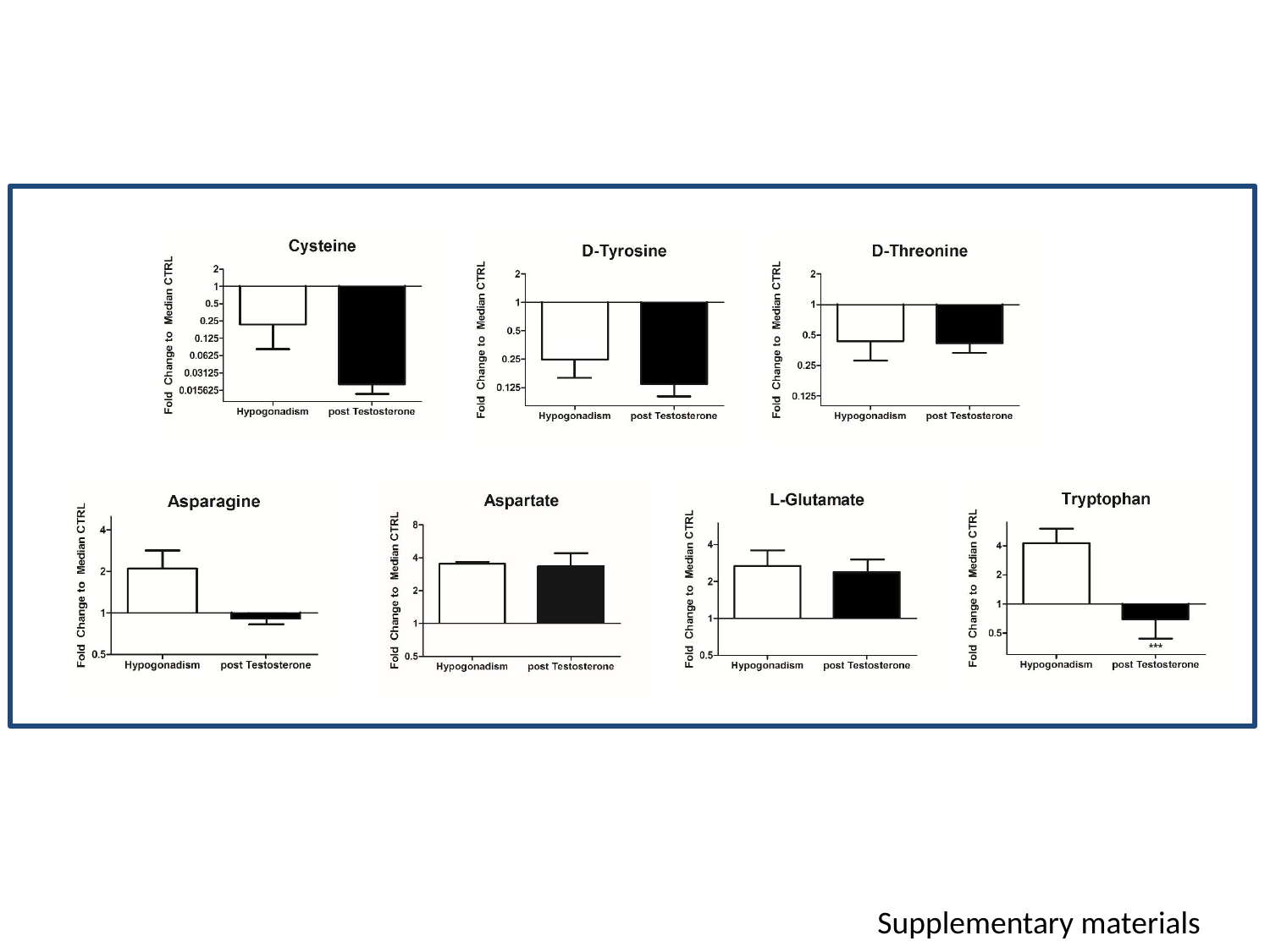

Supplementary materials

Supplement: Supplementary file 1 [file ijms-23-07754-s001.zip › ijms-1768716-supplementary.pptx]
